# Recognizing novel drugs against Keap1 in Alzheimer’s disease using machine learning grounded computational studies

**DOI:** 10.3389/fnmol.2022.1036552

**Published:** 2022-12-06

**Authors:** Nobendu Mukerjee, Khattab Al-Khafaji, Swastika Maitra, Jaafar Suhail Wadi, Punya Sachdeva, Arabinda Ghosh, Rahul Subhash Buchade, Somdatta Yashwant Chaudhari, Shailaja B. Jadhav, Padmashree Das, Mohammad Mehedi Hasan, Md. Habibur Rahman, Ghadeer M. Albadrani, Ahmed E. Altyar, Mohamed Kamel, Mohammad Algahtani, Khlood Shinan, Abdulrahman Theyab, Mohamed M. Abdel-Daim, Ghulam Md. Ashraf, Md. Mominur Rahman, Rohit Sharma

**Affiliations:** ^1^Department of Microbiology, West Bengal State University, Kolkata, India; ^2^Department of Health Sciences, Novel Global Community Educational Foundation, Hebersham, NSW, Australia; ^3^College of Dentistry, The University of Mashreq, Baghdad, Iraq; ^4^Department of Microbiology, Adamas University, Kolkata, India; ^5^Department of Pharmacy, Al-Rafidain University College, Baghdad, Iraq; ^6^Amity Institute of Neuropsychology and Neurosciences, Amity University, Noida, India; ^7^Microbiology Division, Department of Botany, Gauhati University, Guwahati, India; ^8^Department of Pharmaceutical Chemistry, SCES’s Indira College of Pharmacy “Niramay”, Pune, India; ^9^Department of Pharmaceutical Chemistry, Progressive Education Society’s Modern College of Pharmacy, Pune, India; ^10^Center for Biotechnology and Bioinformatics, Dibrugarh University, Dibrugarh, India; ^11^Department of Biochemistry and Molecular Biology, Faculty of Life Science, Mawlana Bhashani Science and Technology University, Tangail, Bangladesh; ^12^Department of Global Medical Science, Wonju College of Medicine, Yonsei University, Wonju-si, South Korea; ^13^Department of Biology, College of Science, Princess Nourah Bint Abdulrahman University, Riyadh, Saudi Arabia; ^14^Department of Pharmacy Practice, Faculty of Pharmacy, King Abdulaziz University, Jeddah, Saudi Arabia; ^15^Department of Medicine and Infectious Diseases, Faculty of Veterinary Medicine, Cairo University, Giza, Egypt; ^16^Department of Laboratory and Blood Bank, Security Forces Hospital, Mecca, Saudi Arabia; ^17^Department of Computer Science, College Computer Science in Al-Leith, Umm Al-Qura University, Mecca, Saudi Arabia; ^18^College of Medicine, Alfaisal University, Riyadh, Saudi Arabia; ^19^Department of Pharmaceutical Sciences, Pharmacy Program, Batterjee Medical College, Jeddah, Saudi Arabia; ^20^Department of Pharmacology, Faculty of Veterinary Medicine, Suez Canal University, Ismailia, Egypt; ^21^Department of Medical Laboratory Sciences, College of Health Sciences, University of Sharjah, Sharjah, United Arab Emirates; ^22^Department of Pharmacy, Faculty of Allied Health Sciences, Daffodil International University, Dhaka, Bangladesh; ^23^Department of Rasa Shastra and Bhaishajya Kalpana, Faculty of Ayurveda, Institute of Medical Sciences, Banaras Hindu University, Varanasi, India

**Keywords:** Alzheimer’s disease, neurodegeneration, QSAR, molecular docking and dynamics simulation, Keap1, oxidative stress, amyloid-beta, phytochemicals

## Abstract

Alzheimer’s disease (AD) is the most common neurodegenerative disorder in the world, affecting an estimated 50 million individuals. The nerve cells become impaired and die due to the formation of amyloid-beta (Aβ) plaques and neurofibrillary tangles (NFTs). Dementia is one of the most common symptoms seen in people with AD. Genes, lifestyle, mitochondrial dysfunction, oxidative stress, obesity, infections, and head injuries are some of the factors that can contribute to the development and progression of AD. There are just a few FDA-approved treatments without side effects in the market, and their efficacy is restricted due to their narrow target in the etiology of AD. Therefore, our aim is to identify a safe and potent treatment for Alzheimer’s disease. We chose the ursolic acid (UA) and its similar compounds as a compounds’ library. And the ChEMBL database was adopted to obtain the active and inactive chemicals against Keap1. The best Quantitative structure-activity relationship (QSAR) model was created by evaluating standard machine learning techniques, and the best model has the lowest RMSE and greatest R2 (Random Forest Regressor). We chose pIC50 of 6.5 as threshold, where the top five potent medicines (DB06841, DB04310, DB11784, DB12730, and DB12677) with the highest predicted pIC50 (7.091184, 6.900866, 6.800155, 6.768965, and 6.756439) based on QSAR analysis. Furthermore, the top five medicines utilize as ligand molecules were docked in Keap1’s binding region. The structural stability of the nominated medications was then evaluated using molecular dynamics simulations, RMSD, RMSF, Rg, and hydrogen bonding. All models are stable at 20 ns during simulation, with no major fluctuations observed. Finally, the top five medications are shown as prospective inhibitors of Keap1 and are the most promising to battle AD.

## Graphical Abstract

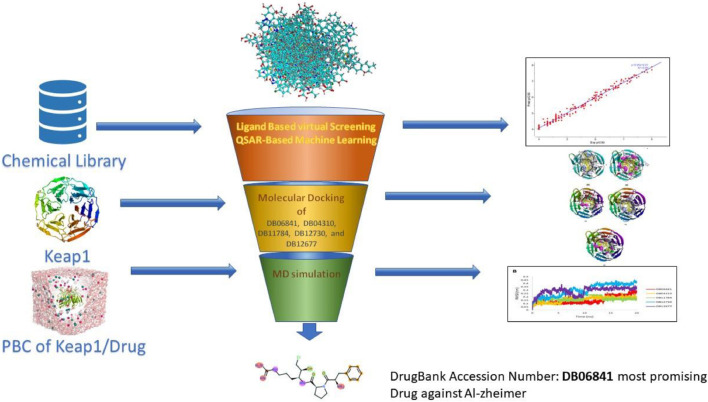

## Introduction

Alzheimer’s disease (AD) is the most common type of dementia and the sixth greatest cause of mortality in Western societies, posing a serious public health threat (Isaacson et al., 2018). Late-onset AD is now recognized to begin decades before a dementia diagnosis, with a long prodromal period that commonly starts in midlife (Frisoni et al., 2011). Preclinical AD is the first stage of this prodromal phase, which has no visible cognitive symptoms but provides a huge possibility for early intervention (Ghosh S. et al., 2022; Sharma et al., 2022). A few individuals with AD will exhibit neuropsychiatric symptoms, also known as behavioral and psychological symptoms of dementia (Sharma et al., 2019). People who have dementia often have AD, which is followed by vascular dementia and a number of other conditions that affect the brain. AD has been hard to tell apart from all the other dementia-causing neuropathology that have come up in recent years, even though there has been a lot of research and new brain imaging technology. Neuropathological disorders and the gradual start of AD are almost impossible to tell apart and diagnose at an early stage because they both happen simultaneously. It’s become more common for people to write about how to separate “AD” from the pathological traits often found in people with the disease, which is called a diagnostic (Hyman et al., 2012). Because a lot of research has shown that many people with AD have neuropathological problems after they die, this distinction between clinical and pathological concepts is correct. Even though modern imaging methods can show amyloid load *in vivo*, it is hard to figure out how much synaptic loss, gliosis, Lewy bodies, neuron loss, granulovacuolar degeneration, and cerebral amyloid angiopathy there is before death. In people with AD, many other illnesses follow up, like Lewy body disease and vascular traumas, that can worsen their cognitive abilities. In light of this, new drugs with antidepressant and neuroprotective properties are urgently needed (Lanoiselée et al., 2017; Sharma et al., 2018, 2021). AD is caused by mutations in the genes that make the amyloid precursor protein, presenilin 2 (PSEN2) and presenilin 1 (PSEN1) (Chiu et al., 2013). To make things even better, the treatment plan should include pharmaceutical and non-pharmaceutical therapies. These could be activities that are physical, social, or even cognitive. It treats mild to moderate cognitive impairment with the drugs sodium valproate and lithium (Moore et al., 2010). There are even some well-known pain relievers that can help prevent oxidative damage (Kaspar et al., 2009; Hane et al., 2017), neurodegeneration, and reduce neuroinflammation, like naproxen and ibuprofen, which are both non-steroidal anti-inflammatory drugs.

There are over 20,000 compounds in triterpenoids, which are secondary metabolites with diverse biological roles. Triterpenoids are from a complex collection of secondary metabolites that contain over 20,000 compounds with various biological functions. Oleanolic acid, betulinic acid, and ursolic acid (UA) are the three most prevalent triterpenoids found in plants, with oleanolic acid being the most abundant. It has been discovered that these chemicals, among other biological effects, exhibit antifungal, anti-HIV, and anti-tumor properties. In addition to being a phytosterol and a pentacyclic triterpenoid, UA has also been shown to have pharmacological activity, which was previously considered the case. It was primarily utilized as an emulsifier in the pharmaceutical, cosmetic, and food sectors, among other places. For this reason, it is regarded harmless; it has been shown to have no adverse effects in mice at dosages of up to 1,000 mg/kg body weight (Kerr et al., 2017) due to its low toxicity. A study published in neurology found that UA can change the monoaminergic system, which may be necessary to prevent mood and cognitive dysfunctions associated with neurodegenerative and mental diseases (Rafi et al., 2020). In 1920, it was discovered in apple epicuticular waxes (Gaulton et al., 2012), which led to its discovery. Recent discoveries have revealed that it can be found in the wax coverings of various fruits, including prunes, pears, cranberries, bilberries, and olives (Gaulton et al., 2012; Rafi et al., 2020), as well as the medicinal plants *Eriobotrya japonica, Rosmarinus officinalis, Arctostaphylos uva-ursi*, and *Ocimum sanctum* (Yap, 2011; Mukerjee et al., 2022).

**FIGURE 1:**
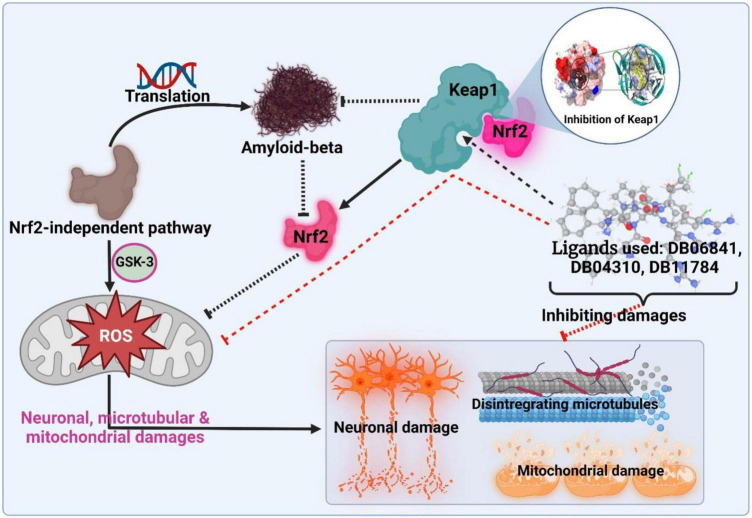
Schematic presentation of the active inhibition. Adopted from BioRender.com.

Computer-aided drug design (CADD), which has the potential to reduce the amount of effort, time, and money spent on medication research, has become increasingly essential in recent years (Mukerjee et al., 2022). Artificial intelligence and computational technologies are used to screen and analyze millions of compounds in libraries to find the most promising drugs. This project aims to use artificial intelligence and computational methods to find possible and safe inhibitors of Keap1. As a result, for the objective of screening keap1 inhibitors, we built a QSAR based on machine learning. The first and most critical step was to create an effective QSAR model by choosing the best machine learning technique from a selection of options. Based on their R2 and RMSE scores, the authors compared and evaluated forty-one machine learning methods. After that, the best model (with the highest R2 and lowest RMSE) was used to calculate pIC50 and screen the library that had been gathered. The compounds library was created based on the structural similarity of the UA compound. The medicines with the highest pIC50 values were identified as the most promising treatments against Keap1 when predicted pIC50 values for the complete chemical library were compared. We have inhibited Keap1 using novel phytochemical UA in this study using machine learning and molecular dynamics simulation approaches (as illustrated in Figure 1).

## Materials and methods

### United States food and drug administration-approved drugs library

The compounds which have a similar structure to UA were screened in the drug bank database by using the SWISS similarity tool.^1^

### Dataset curation and machine learning model training

**FIGURE 2:**
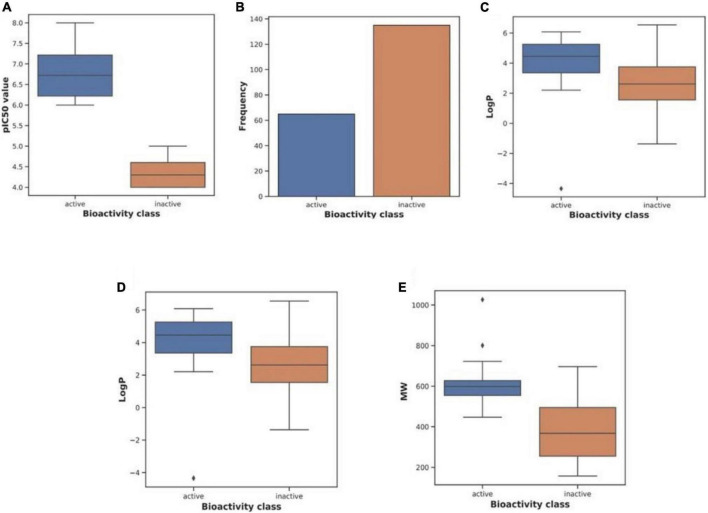
Characteristics of the curated dataset. Adopted from BioRender.com.

We used CHEMBL (Wang et al., 2016) to acquire the dataset, which contains small compounds with experimentally validated IC_50_ values against Keap1. To train classification models, known experimental activity levels (IC_50_) were used and activity labels were assigned based on the activity values. The training activity cut-off was established at 1,000 nM; if IC50 = 1,000 nm is active, either is inactive. Figure 2 shows more information about the curated dataset, which has 65 active and 174 inactive. The dataset’s PubChem fingerprints were then calculated using the open-source software “PaDEL for descriptor calculation” (Ravindranath et al., 2015). The typical algorithms were then evaluated and compared using lazyPredict. Based on the root mean squared error (RMSE) and R-squared, the optimal algorithm was chosen (R2). The entire dataset was utilized as a training set before being used as a test set.

### Molecular docking

The 3D structures of Keap1 downloaded protein data bank (PDB ID: 4IFN). The top five drugs obtained from ML-based QSAR screening were prepared by Checm3D software. The AutodockFR (ADFR) program (Al-Khafaji and Taskin Tok, 2020b) and AutoGridFR (AGFR version 1.0) (Al-Khafaji and Taskin Tok, 2021) programs are used in the execution of the molecular docking of ligands in the active site of Keap1 which are capable of establishing configuration file which contains the data for running controlled flexible docking by identifying the residues of the complex’s binding site. Autogrid maps estimated with a default grid map were set on the position of reference ligands at (40, 40, 40) box spacing of 0.375A0. ADFR’s presumptive parameters enable the ligand to reach buried grooves (Al-Khafaji and Taskin Tok, 2020a,^2021^). ADFR uses the scoring function of Autodock but is customized for flexible receptors (Shukla et al., 2019; Ghosh A. et al., 2022). The docking was implemented using default search settings.

### Molecular dynamic simulation

Dynamics simulations are of vital interest to investigators in the field of computer-aided drug discovery (Yap, 2011; Zoete et al., 2011). Thus, we are using molecular dynamics simulations to supply us with information about conformational stability (Bjelkmar et al., 2010; Shukla et al., 2019). Like these technologies, molecular dynamics simulation consists of a set of standard protocols of GROMACS 2018.1 and an implementation. We implemented these protocols holo forms of Keap1, with Charmm27 force field for all atoms (Darden et al., 1993; Bjelkmar et al., 2010; Yap, 2011). For the first step of molecular dynamics, we employed the Swiss PARAM to create the topology file of drugs (Zoete et al., 2011). The holoforms of Keap1 were placed and cantered in a cubic cell unit with at least 0.1 nm distance from box edges. Then, we chose three-point transferable intermolecular potential (TIP3P) that is used as a solvent for our protein-ligand complexes. And if complexes were charged, we neutralized the complexes by adding opposite ions either sodium ions or chloride ions. An appropriate way to proceed with this molecular dynamic simulation is to use the steepest descent algorithm threshold value of 1,000 kJ/mol nm for minimization. Both the NVT and NPT ensembles of 0.1 ns were used with the position constraint on the protein molecules for controlling pressure at 1 atm and temperature at 300 K. In the next step, to assess the impact of electrostatic interactions on the diversified behaviors of complexes, we utilized the Particle Mesh Ewald summation. Then, we implemented a 20 ns MD simulation for all systems. The MD trajectories were analyzed by using the RMSD, RMSF, Rg, hydrogen bonding, and principal component analysis (PCA).

## Results

### Inhibitory activity prediction via machine learning model

**FIGURE 3:**
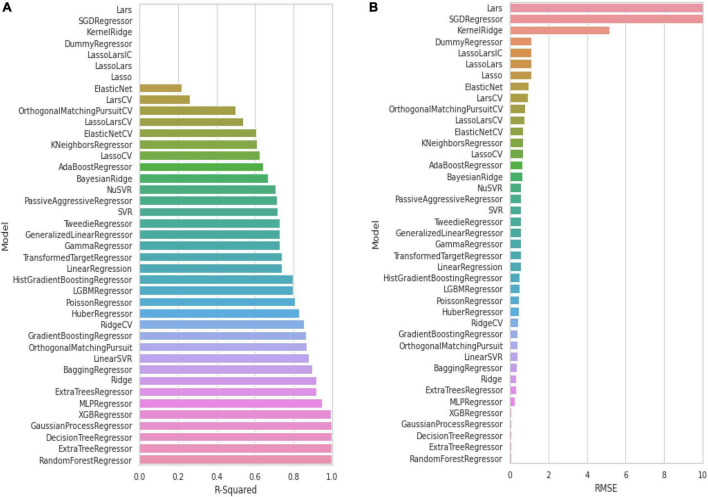
Evaluation of common machine learning algorithms via using: **(A)** R-squared and **(B)** RMSE. Adopted from BioRender.com.

**FIGURE 4:**
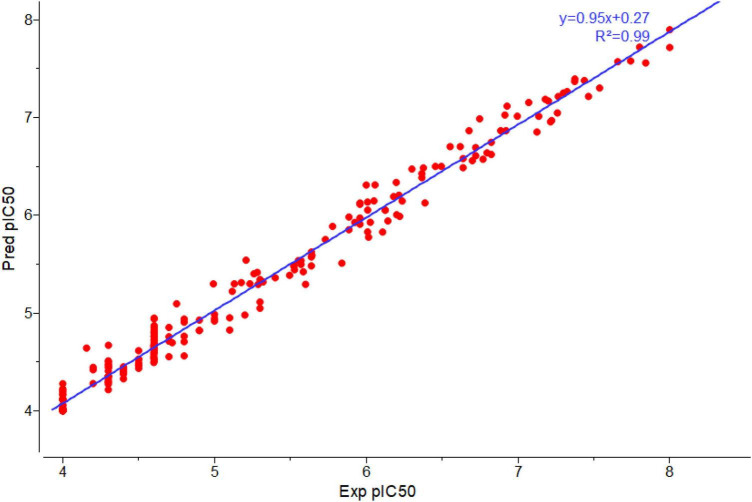
Plot correlation between experimental and predicted pIC50 by using Random Forest-regressor. Adopted from BioRender.com.

To build a model which can be used to determine the inhibitory activity (pIC50) against Keap1, we compared common machine learning algorithms. The ability of these algorithms to build an efficient model was assessed via (R2) and RMSE, from benchmarking the common algorithms. The benchmarks of the R2 and RMSE indicated that the Random Forest Regressor (Figure 3) can be used as an efficient model to predict pIC50. Further, the correlation between experimental and predicted pIC50 is presented in Figure 4. Then this model was used to predict pIC50 values (as shown in Supplementary Data Sheet 1). It can be observed that the UA has a pIC50 of 5.553, whereas the top five pIC50 were 7.1, 6.9, 6.8, 6.77, and DB06841, DB04310, DB11784, DB12730, and DB12677, respectively.

### Molecular docking analysis

**FIGURE 5:**
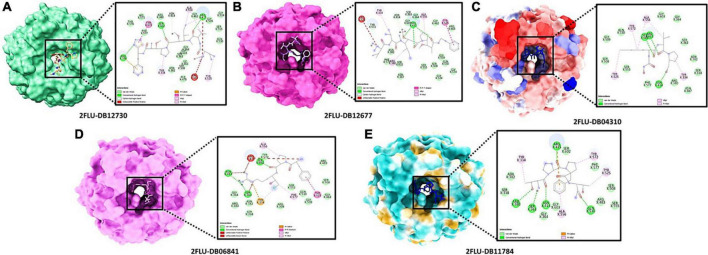
Describes the docking pose of Pdb: 4IFN and our 5 selected drug molecules.

The best confirmation was displayed as a docking complex, according to the results, after completing a molecular docking analysis of the Keap1 protein with our five-hit ligands: DB12730, DB12677, DB12310, DB06841, and DB11784 in the ADFR output. For usage with the MGL 1.5.6 suite, the receptors and ligands were stored in pdbqt format. By putting the command into the command prompt, ADFR was launched. It was configured to use exhaustiveness of eight and a grid point spacing of 0.275 by default. PyMol and the Discovery studio visualizer 2021 were used to investigate the output files in pdbqt format and they were found to be accurate. Using a co-crystal ligand, we were able to verify and improve the ligand binding. It was made by mixing 48 hydrogen bonds, one of which was rotatable, with Kollman and Gastieger charges, and then adding them to the resulting combination. Finally, the pdbqt format was used to hold both the receptor and ligand molecules. To create a grid box, the values *X* = –1.655, *Y* = 57.005, and *Z* = 133.83 were multiplied by 0.275. The Genetic Algorithm was used to perform docking studies on the protein-ligand combination (GA). A total of 3,000,000 evaluations and 27,000 generations of GA were used with parameters such as population size of 100, evaluation number of 150, and evaluation number of 3,000,000. Additional docking experiments were performed using the Lamarckian genetic algorithm (LGA) to find the lowest free energy of binding for the protein-ligand complex (G). The free energy of binding (G) of 4IFN with the DB12730 complex was –7.658 kcal/mol, the inhibitory concentration (Ki) was 2.55 mM, the ligand efficiency was –0.26, the total internal energy was –0.45 kJ/mol, and the torsional energy was 0.3 kcal/mol; the inhibitory concentration (Ki) was 2.55 mM, the ligand efficiency was –0.26, the total 4IFN demonstrated free energy of binding (G) of –8.941 kcal/mol in the presence of the DB12677 complex; 4IFN demonstrated free energy of binding (G) of –7.974 kcal/mol in the presence of the DB12310 complex; 4IFN demonstrated free energy of binding (G) of –7.7558 kcal/mol in the presence of the DB06841 complex; and 4IFN demonstrated free energy of binding (G) of the DB11784 complex. The essential residues involved in the creation of the binding pocket surrounding five distinct ligands (DB12730, DB12677, DB12310, DB06841, and DB11784) are depicted in Figures 5A–E.

### Dynamic simulation analysis

**FIGURE 6:**
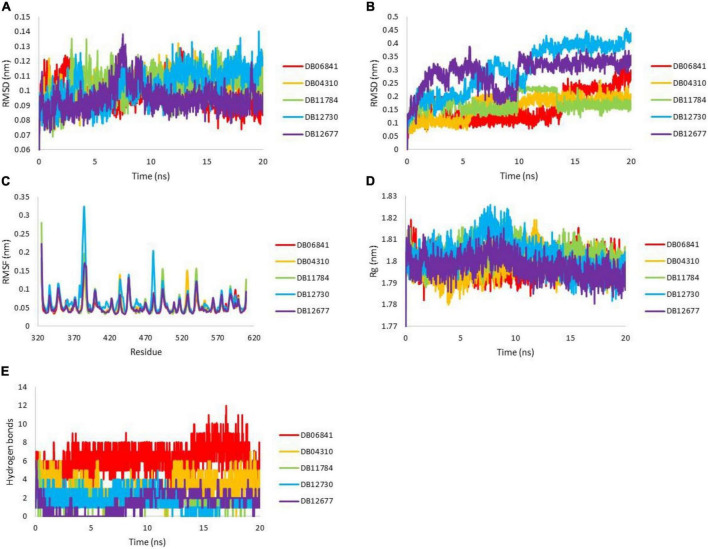
MD simulation analysis for generated trajectories for Keap1 holo forms: **(A)** RMSD for Keap1 backbone atoms, **(B)** RMSD for ligand atoms, **(C)** RMSF for backbone atoms of Keap1 residues, the **(D)** radius of gyration for keap1 atoms and **(E)** hydrogen bonds between the drugs with Keap1. Adopted from BioRender.com.

To understand the dynamic behavior of the Keap1 when it is bound to one of DB06841, DB04310, DB11784, DB12730, and DB12677 during 20 ns MD simulations. The root means square deviation (RMSD), the root means square fluctuation (RMSF), radius of gyration (Rg), and Hydrogen bonding during 20 ns. The RMSD values of the drug-bound Keap1 backbone revealed that all drug-bound Keap1 backbones fluctuated between 0.07 and 0.14 nm (Figure 6A). The RMSD values for holo Keap1 showed approximately similar changes in the Keap1 backbones from the starting structures. In deep inspection, the average RMSD values were 0.0992, 0.1036, 0.1032, 0.1033, and 0.0949 for Keap1 backbone bound to DB06841, DB04310, DB11784, DB12730, and DB12677, respectively. Further, the RMSD fluctuations of the inspected drugs were analyzed also to evaluate the dynamics and stabilities of these drugs inside the Keap1’s binding site. It can be observed that DB06841, DB04310, and DB11784 had restricted fluctuations (RMSD less than 0.3 nm during the whole MD simulation) inside the binding site of Keap1 (Figure 6B) and their overall average RMSD values were 0.1587, 0.1622, 0.1736 nm, respectively. Another significant aspect of Md simulation is the RMSF, which is the average fluctuation of each residue used for assessing the flexibility of protein during a simulation. The RMSF values of the top five drugs bound Keap1 are plotted in Figure 6C. From Figure 6C, only RMSF for DB12730-bound Keap1 had higher flexibility for the 330–480 aa region. The overall average RMSF values were 0.0541, 0.0574, 0.0576, 0.0663, and 0.0555 for DB06841, DB04310, DB11784, DB12730, and DB12677, reactively. On the other hand, despite these findings of the impact of DB06841, DB04310, DB11784, DB12730, and DB12677 on the dynamic behavior of Keap1, we used Rg as a tool to analyze the effect of these drugs on the compactness of Keap1. Rg values of drug-bound Keap1 complexes were shown in Figure 6D. Rg results revealed that there are no significant changes in the Keap1 compactness when it is bound to one of these drugs, whereas the average of Rg values were 1.7984, 1.7976, 1.8028, 1.8025, and 1.7976 for DB06841, DB04310, DB11784, DB12730, and DB12677, respectively.

The strength of binding of these drugs toward Keap1’s binding site was evaluated by measuring the hydrogen bonds between the five drugs and Keap1’s active site. The plot of hydrogen bonds between the drugs and Keap1 is shown in Figure 6E. It can be noticed from Figure 6 that DB06841 has the highest number of hydrogen bonds and DB04310 comes second in this rank. The average hydrogen bonding values was 6.1759, 3.2108, 1.9280, 1.7881, and 1.5337 nm, of DB06841, DB04310, DB11784, DB12730, and DB12677, respectively. The Md simulation findings help us to understand how DB06841 and DB04310 have the highest predicted pIC50 values.

### Principal component analysis

**FIGURE 7:**
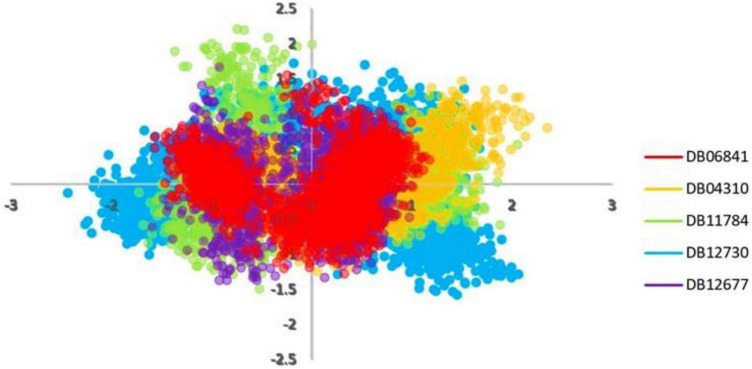
PCA analysis for MD simulation trajectories. Adopted from BioRender.com.

Principal component analyses were executed for the MD trajectories of holo Keap1. To plot PCA or essential dynamics both PC1 and PC2 were plotted together to visualize collective motions (Figure 7). It can be seen that the range of the PCA values for DB06841 is smaller than another drug (Figure 7), indicating more restricted motions of the Keap1 backbone atoms, probably due to a greater number of intermolecular hydrogen bonds formed between the Keap1 and the DB06841. This suggests that the DB06841 decreased the flexibility of Keap1 due to its binding strength inside the binding site also the dynamic behavior was so conserved during the whole MD simulation. Thus, PCAs indicate that the overall motions of the Keap1 complexed with the DB06841 are more conserved compared to the studied drugs.

## Discussion

The goal of this study was to understand more about Keap1 and its role in Alzheimer’s disease so that we could create better treatments. Amyloid-beta-induced neuronal damage can be averted by inhibiting Keap1, a negative regulator of Nrf2. We investigated many machine learning approaches and used the one that showed to be the best successful at predicting inhibitory activity (pIC50) against Keap1. A small number of treatments have shown promise in human trials, but all have severe limitations and address only a subset of the factors that lead to the development of Alzheimer’s disease. By comparing several common machine learning techniques, the best prospective quantitative structure-activity relationship (QSAR) model was developed; this model has the lowest RMSE and highest R2 values. Our top five drugs (DB06841, DB04310, DB11784, DB12730, and DB12677) were chosen based on the QSAR study’s predicted pIC50 values (7.091184, 6.900866, 6.800155, 6.768965, and 6.756439). The binding location of Keap1 was also used to dock the molecules of the five most commonly prescribed drugs. Following that, molecular dynamics simulations, RMSD, RMSF, Rg, and hydrogen bonding were used to assess the structural stability of the potential drugs. At 20 ns, none of the models exhibit any discernible oscillations. Finally, five drugs with the highest promise as Keap1 inhibitors in the fight against Alzheimer’s disease are offered.

## Conclusion

**FIGURE 8:**
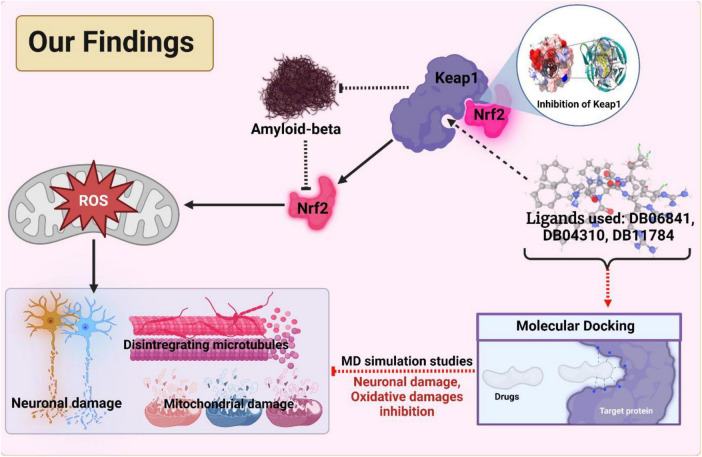
The outcomes and findings in our research show the active inhibition of the target protein. Adopted from BioRender.com.

This study was done to investigate the potential drug against AD via targeting Keap1. A specific inhibition of Keap1, which is a negative regulator of Nrf2, can help in the prevention of neuronal toxicity caused by amyloid-beta. To build a model which can be used to determine the inhibitory activity (pIC50) against Keap1, we compared common machine learning algorithms and implement the best model (Random Forest regressor). We have found five chemical compounds with generic names: 2-[(Formyl-Hydroxy-Amino)-Methyl]-Heptanoic Acid [1-(2-Hydroxymethyl-Pyrrolidine-1-Carbonyl)-2-Methyl-Propyl]-Amide (DrugBank ID: DB04310, Chemical Formula: C19H35N3O5), [(2R)-1-[(2S)-2-[[(2S,3S)-1-Chloro-6-(diamino methylidene amino)-2-hydroxyhexan-3-yl]carbamoyl]pyrrolidin-1-yl]-1-oxo-3-phenylpropan-2-yl]a zanium (DrugBank ID: DB06841, Chemical Formula: C21H34ClN6O3), NRX-1074 (DrugBank ID: DB11784, Chemical Formula: C25H37N5O6), Soblidotin (DrugBank ID: DB12677, Chemical Formula: C39H67N5O6), and Dolastatin 10 (DrugBank ID: DB12730, Chemical Formula: C42H68N6O6S); these compounds are small-molecule types showing the highest pIC50 values, and then we run on molecular docking for these drugs against Keap1 target. Also, these drugs showed very stable interactions with Keap1. To examine the dynamic stabilities of nominated drugs/Keap1, further, we run the MD simulation and PCA analysis for MD trajectories. The Md simulation and PCA analysis revealed that DB06841, [(2R)-1-[(2S)-2-[[(2S,3S)-1-Chloro-6-(diaminomethylideneamino)-2-hydroxyhexan-3-yl] carbamoyl]pyrrolidin-1-yl]-1-oxo-3-phenylpropan-2-yl]azanium, can act as most promising drug for preventing neurodegeneration associated with the accumulation of amyloid-beta (as illustrated in Figure 8).

## Data availability statement

The original contributions presented in this study are included in the article/Supplementary material, further inquiries can be directed to the corresponding authors.

## Author contributions

NM and KA-K contributed to the conceptualization. KA-K, NM, SM, AG, RB, and SC designed and carried out the experimental procedures. NM, KA-K, JS, AG, GA, SJ, and MMR did the analysis. NM, MR, PS, SM, MA, and AT did the manuscript preparation. AG, NM, GMA, MK, MA, KS, MA-D, MR, and RS edited the manuscript. All authors contributed to the article and approved the submitted version.

## Footnotes

1
http://www.swisssimilarity.ch
